# Epidemiology of burns in pediatric patients of Beijing City

**DOI:** 10.1186/s12887-016-0686-7

**Published:** 2016-10-18

**Authors:** Shujun Wang, Dawei Li, Chuanan Shen, Jiake Chai, Hongjuan Zhu, Yanlu Lin, Congying Liu

**Affiliations:** Department of Burns and Plastic Surgery, the First Affiliated Hospital of Chinese PLA General Hospital, Beijing, 100048 China

**Keywords:** Burn, Pediatric, Epidemiology, Emergency medicine, Accident prevention, Wound and injury

## Abstract

**Background:**

This study aimed to assess the epidemiological characteristics of pediatric burns in Beijing City.

**Methods:**

This was a retrospective study of pediatric patients (*n* = 400) admitted to four burn centers in Beijing City between June 2010 and May 2011. Burn severity was determined according to total body surface area (TBSA) percentage and degree. Patients were followed up for one year. Multivariate analyses were carried out to determine the factors (burn etiology, time and place of injury, living conditions, hospital type, first-aid treatment methods, and parent/guardian knowledge of burns, educational level, occupation) affecting burn properties (severity and pigmentation/scar).

**Results:**

191/400 (47.8 %) patients were aged 2-3 years, and scalding was the leading etiology (355/400, 88.8 %). Burn incidence peaked in May (14.8 %), at 10:00-12:00 and 17:00-20:00. Most burn events occurred indoors (272/400, 68.0 %), especially in the kitchen (180/400, 45.0 %). Roughly half of them involved head and neck; 188 (47.0 %) patients had mild burns, 140 (35.0 %) moderate, 44 (11.0 %) extensive, and 28 (7.0 %) critical burns; 184 (46.0 %) patients were treated only with cold-water rinsing or compress; 120 (30.0 %) received no first aid. Only 32 (8.0 %) patients visited a specialized institution. 164 patients underwent surgery. Hospitalization lasted for 14.8 ± 8.1 days. Independent risk factors for burn severity were occurrence month, living conditions, occupation of the mother, and first aid. 288 (72.0 %) patients developed pigmentation and scar within a year while no independent risk factors was observed.

**Conclusions:**

Pediatric burns often occurred indoors, especially in the kitchen, and a substantial proportion receives no first aid.

**Electronic supplementary material:**

The online version of this article (doi:10.1186/s12887-016-0686-7) contains supplementary material, which is available to authorized users.

## Background

The incidence of burns is about 1 % in the world [1] and 0.79 % in children [2]. Pediatric burns may not only cause life-long disability, but also affect the mental health and quality of life of their families, imposing a socioeconomic burden [3].

While the incidence of pediatric burns in Chinese hospitals has decreased in the past forty years [4–7], scalds still has a high incidence in China, which may be somewhat related to eating habits, as most Chinese meals are served hot (boiled, steamed, or deep fried) [8–10]. Currently, children account for 21.6-54.7 % of hospitalized burn patients in China [7]. The socioeconomic status is increasing rapidly in China and has profound impacts on lifestyle and health [11], and there is a lack of data about the epidemiology of burns in the pediatric population of fast developing cities in China. The sixth Chinese census in 2010 reported that 1,687,000 children between 0 to and 14 years of age reside in Beijing city, while more than 200 million children were recorded in China (http://www.nhfpc.gov.cn/htmlfiles/zwgkzt/ptjnj/year2011/index2011.html) [12]. Such a large population of children residing in such a fast developing city warrants further study of the epidemiological profile of pediatric burns.

Therefore, the aim of the present study was to assess the epidemiology of pediatric burns in the unique city of Beijing.

## Methods

This retrospective study evaluated the medical data of all pediatric burn patients treated between June 2010 and May 2011 at four major Beijing hospitals specialized in burn trauma, or general hospitals with a burn trauma department: Department of Burns & Plastic Surgery (The First Affiliated Hospital of Chinese PLA General Hospital), Department of Burns Surgery (The second Artillery General hospital of Chinese PLA), Department of Burns Surgery (Beijing Children’s Hospital), and Department of Burn & Plastic (You An Men Hospital of Association of Beijing Hospital). To be included, patients had to meet the following criteria: (1) <14 years of age; (2) admission to hospital within 24 h of burn injury; (3) being alive on discharge; and (4) completion of follow-up telephone interviews. This study was approved by the Ethics Committee of the participating hospitals. Written informed consent was obtained from all children’s parents and guardians for the phone interview follow-up.

The pediatric patients were grouped into four age categories: (1) infants (≤1 year); (2) toddlers (2 to 3 years); (3) preschoolers (4 to 7 years); (4) school age kids (8 to 14 years). According to standards of care for pediatric burns formulated by the Chinese Burn Association [13], the severity of the pediatric burns was graded as follows: mild (<5 % of total body surface area [TBSA] and no third degree burns); moderate (between 5-15 % of TBSA or third degree burn <5 % of TBSA); extensive (between 15–25 % of TBSA or third degree burn between 5 and 10 % of TBSA); and critical, (>25 % of TBSA or third degree burn >10 % of TBSA). The burn area was estimated by two attending physicians at admission to the Burn Department, according to the Rule of Nines and Rule of Palm [13]. During hospitalization, a self-made questionnaire was filled by the parents or guardians, with any unclear item explained on the spot by the attending physicians. This questionnaire included patient demographic and clinical characteristics such as burn etiology, time and place of injury, living conditions, hospital type and methods of first-aid treatment, and parent/guardian knowledge of burns, educational level, and occupation. TBSA, the anatomical areas affected, and surgery duration were reviewed from medical records. Follow-up was carried out by outpatient visits and telephone a year post-discharge to assess the wound condition, pigmentation, and scaring. The presence of pigmentation or scar was recorded as yes or no, based on simple descriptions provided to the parents/guardians.

### Statistical analysis

Continuous data were presented as mean ± standard deviation. Categorical variables were presented as figures and analyzed using the Fisher’s exact test. An ordinal logistic regression analysis (backward method) was performed to determine the factors independently associated with burn properties (burn severity and burn pigmentation as outcomes); covariates initially entered in the model were burn etiology, time and place of injury, living conditions, hospital type, first-aid treatment methods, and parent/guardian knowledge of burns, educational level, and occupation. Statistical analyses were carried out with SPSS 13.0 (SPSS Inc., Chicago, IL, USA). *P* < 0.05 was considered statistically significant.

## Results

### Demographic characteristics

**Fig. 1:**
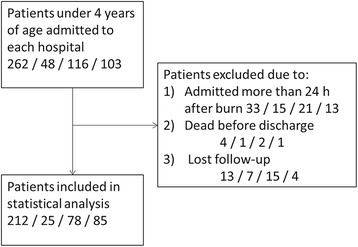
Patient flowchart. The numbers represent the numbers of patients seen at the four hospitals

Between June 2010 and May 2011, 529 patients aged between 0 and 14 years were admitted to the burn departments of four major hospitals (262, 48, 116 and 103 patients, respectively). Of these, 400 pediatric patients were included in this study (212, 25, 78, and 85 patients, respectively) according to the inclusion criteria (Fig. 1). Children <1 year old accounted for 102 (25.5 %) of the 400 pediatric patients; 2 to 3, 4 to 7, and 8 to 14 years old children comprised 191 (47.8 %), 86 (21.5 %), and 21 (5.3 %) of the 400 included patients, respectively. A male to female ratio of 1.3:1 was observed.

### Burn etiology and temporal distribution

**Fig. 2:**
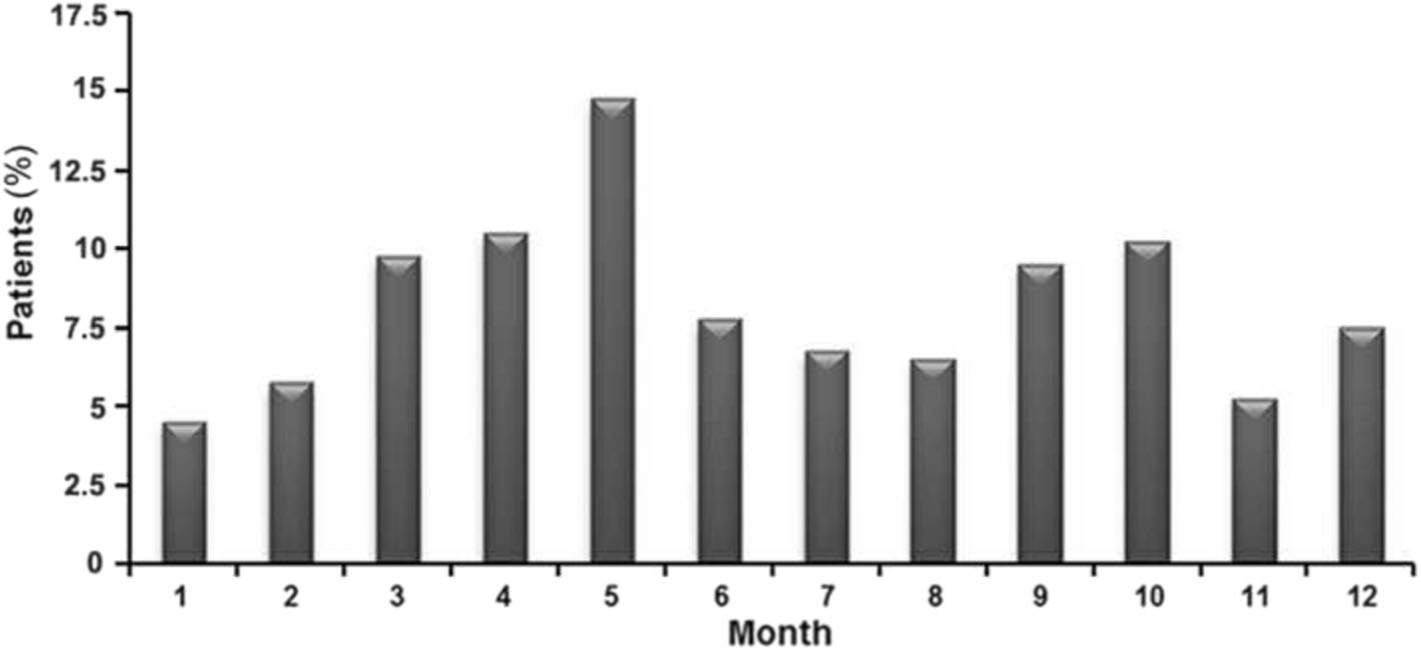
Mean monthly distribution of burns

**Fig. 3:**
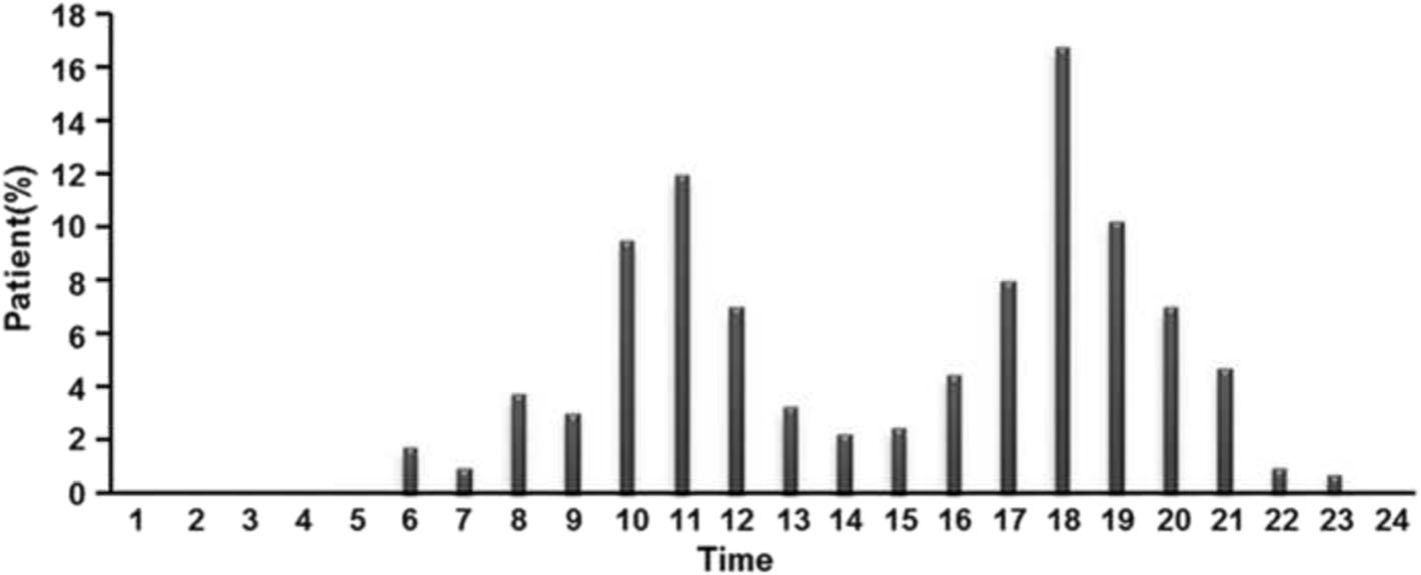
Mean daily distribution of burns

The most common etiology was scalding (355/400, 88.8 %), followed by flame (34/400, 8.5 %), electricity (5/400, 1.3 %), chemicals (3/400, 0.8 %), and others (3/400, 0.8 %) (Table 1). As for temporal distribution, burn events occurred most frequently in May (*n* = 59, 14.8 %), followed by April (*n* = 42, 10.5 %), October (*n* = 41, 10.3 %), and March (*n* = 39, 9.8 %) (Fig. 2). Burn injuries occurred most frequently between 17:00-20:00 (*n* = 168, 42.0 %) and 10:00-12:00 (*n* = 114, 28.5 %) (Fig. 3).

**Table 1:** Distribution of burns by etiology and age

Age (years)	Scald *N* = 355	Flame *N* = 34	Electrical *N* = 5	Chemical *N* = 3	Others *N* = 3
0-1	92 (90.2 %)	8 (7.8 %)	1 (1.0 %)	1 (1.0 %)	0 (0 %)
2-3	174 (91.1 %)	13 (6.7 %)	2 (1.1 %)	0 (0 %)	2 (1.1 %)
4-7	74 (86.0 %)	7 (8.1 %)	2 (2.3 %)	2 (2.3 %)	1 (1.2 %)
8-14	15 (71.4 %)	6 (28.6 %)	0 (0 %)	0 (0 %)	0 (0 %)

Statistical analysis of the population by age: *χ* ^2^ = 8.210 and *P* = 0.042 for scald, *χ* ^2^ = 11.653 and *P* = 0.009 for flame. The percentages were calculated in the age subgroups

### Living conditions

The majority of patients (*n* = 260, 65.0 %) lived in urban areas. A total of 264 patients (66.0 %) lived in bungalows (simple and primitive houses), and 136 in apartments. Most patients (*n* = 272, 68.0 %) were injured at home, with 181 (45.3 %), 81 (20.3 %), and 12 (3 %) in the kitchen, bedroom, and hall, respectively; patients were also burned at the kindergarten (*n* = 11, 2.8 %) and in public places (*n* = 115, 28.8 %).

### Burn areas, severity, and treatment before hospitalization

The most frequently injured anatomical area was head and neck (*n* = 214; 53.5 %), followed by lower (*n* = 209; 52.3 %) and upper (*n* = 192; 48.0 %) extremities. Mild injuries were the most common (*n* = 188, 47.0 %), followed by moderate (*n* = 140, 35 %), severe (*n* = 44, 11.0 %), and extremely severe (*n* = 28, 7.0 %) ones. As for treatment before hospitalization, 184 pediatric patients (46.0 %) had injuries treated only with cold-water rinsing or compress; 68 (17.0 %) had clothes cut away, 28 (7.0 %) received skin application of “folk nostrum” (any traditional folk medicine; it is a collection of traditional folk medicine that includes wet compresses and/or unguents, mostly based on the traditional Chinese medicine), and the remaining 120 (30.0 %) did not receive any first aid. After burns, 248 patients (62.0 %) were brought to general hospitals, 108 (27.0 %) to community hospitals, and 32 (8.0 %) to hospitals with a specialized burn department; 12 (3.0 %) of patients were taken to other hospital types. At the hospital, 228 patients (57.0 %) had wounds disinfected and dressed; 76 (19.0 %) had theirs rinsed with cold water, 60 (15.0 %) received exposure treatment, and 36 (9.0 %) were treated with other approaches.

### Characteristics of parents/guardians

Among the parents/guardians of the injured children, 96 (24.0 %) and 120 (30.0 %) fathers/male guardians had high school and college degrees, respectively; these numbers were 82 (20.5 %) and 118 (29.5 %) for mothers/female guardians. Among all parents/guardians, 110 (27.5 %) and 105 (26.3 %) fathers/male guardians were farmers and office workers, respectively; 89 (22.3 %) and 90 (22.5 %) of mothers and female guardians were farmers and office workers; finally, 62 (15.5 %) and 54 (13.5 %) of fathers/male guardians and mothers/female guardians were laborers, respectively.

Nearly half (*n* = 188, 47.0 %) of caregivers had no knowledge of burn injury prevention and treatment; 164 (41.0 %) claimed to have limited knowledge, and only 48 (12.0 %) had a relatively good understanding thereof.

### Medical care and follow-up results

The majority of pediatric patients (*n* = 236, 59.0 %) underwent no surgery, while 132 (33.0 %) and 32 (8.0 %) had one and multiple surgeries, respectively. Overall, among the patients that underwent surgery, the mean number of surgeries per patient was 1.24 ± 0.54 and included debridement and/or skin grafting; the timing of the surgeries was based on the decision of the physicians. Hospitalization durations were 0–7, 8–14, 15–21, and >21 days for 76 (19.0 %), 168 (42.0 %), 72 (18.0 %), and 84 (21.0 %) of patients, respectively. The majority (*n* = 288, 72.0 %) of patients developed pigmentation and scaring after wound healing.

### Independent risk factors of burn severity and outcome

The ordinal logistic regression analysis indicated that occurrence month (OR = 1.70, 95 % CI: 1.53–1.89, *P* < 0.001), living conditions (OR = 3.60, 95 % CI: 1.82-7.13, *P* < 0.001), occupation of the mother (OR = 1.35, 95 % CI: 1.07-1.71, *P* = 0.012), and first aid (OR = 1.52, 95 % CI: 1.15-2.01, *P* = 0.003) were independently associated with burn severity (Table 2).

**Table 2:** Ordinal logistic regression analysis of risk factors for pigmentation and scar

		95 % CI		
	Adjusted OR	Lower	Upper	*P*
Gender	1.026	0.525	2.004	0.940
Age	0.923	0.794	1.069	0.282
Burn etiology	0.424	0.231	0.776	0.006
Occurrence months	1.702	1.531	1.888	<0.001
Occurrence hours	1.074	0.993	1.159	0.071
Living conditions	3.597	1.816	7.129	<0.001
House type	1.858	0.873	3.954	0.108
Occurrence location	1.009	0.828	1.227	0.934
Occupation of father	0.927	0.713	1.205	0.575
Occupation of mother	1.352	1.069	1.710	0.012
Educational attainment of father	0.726	0.426	1.236	0.239
Educational attainment of mother	0.889	0.546	1.452	0.641
Knowledge of burn prevention	1.040	0.611	1.770	0.885
First aid	1.521	1.148	2.014	0.003

*OR* odds ratio, 95%*CI* confidence interval

## Discussion

To analyze the epidemiological characteristics of pediatric burns in Beijing City, we retrospectively analyzed the demographic and clinical characteristics of 400 pediatric patients admitted to four major burn centers in Beijing City between June 2010 and May 2011. Multivariate analysis indicated that occurrence month and location, housing type, and parent/guardian occupation, educational level, and knowledge of burn prevention were independent risk factors for burn severity. Although there was no statistically significant risk factor for treatment outcome, the type of hospital visited showed a tendency towards an association.

The majority of enrolled pediatric patients were below the age of 3, consistent with a previous study that assessed burn patients in Sichuan Province (southwest China) in 2012, and a large study of the data from the Chinese Trauma Databank between 2001 and 2007 [4, 14]. Roughly half of patients were between 2 and 3 years old, when the motor abilities are limited and characterized by uncoordinated movements, slow reflexes, and partial lack of protective reactions [15]. As a result, these children are particularly vulnerable to burns. Head, neck, and lower and upper extremities were most frequently involved. These body parts are more susceptible to burns because they are more likely to be exposed. In younger children, safety responses are less coordinated, indicating that their guardians need to intervene to reduce the risk of burn.

More male than female pediatric patients were admitted, despite the 2010 census reporting a nearly balanced gender ratio in this age range (male to female ratio of 105.2:100) [12], suggesting that burns are more prevalent in boys than girls [11], corroborating other studies of pediatric burns [16–18]. The leading cause of pediatric burns was scalding, accounting for 88.8 % of all cases, in agreement with previous reports showing that scalds are a common cause of burn in the home, with child age and gender affecting the risk of burns [2, 4, 16–20]. Nevertheless, it should be noted that the 88.8% of burns caused by scalding observed in the present study is markedly higher than values reported previously, which are usually <50 %. This may be somewhat related to eating habits, as most Chinese meals are served hot and prepared by boiling, steaming, and deep frying [8–10]. It is known that burn injury in resource-poor settings is rather complex; indeed, the World Health Organization (WHO) estimates that burns kill more than 310,000 individuals each year, including 95 % in low and middle income countries (LMIC), with scalding being the most prominent etiology in children [19].

In the present study, many pediatric patients required extensive medical care. Indeed, 41 % underwent one or more surgeries, with 39 % being hospitalized for over two weeks. In addition, 72 % of the pediatric patients developed post-surgery pigmentation and scars, which combined with movement dysfunction could cause irreparable damage to the injured children and their families.

In the present study, pediatric burns occurred most frequently between 10:00-12:00 and 17:00-20:00, with approximately 50 % of all events occurring in the kitchen, suggesting that they likely occurred during meal preparation or while cleaning up after serving food. Pediatric burn incidence gradually rose from January and peaked in May. These trends were also observed in an 11-year study in Datong, China [21], and may be attributed to seasonal variations of climate. As it gets warmer, children may wear less clothing, possibly exposing more skin areas to burns. During these months, however, warm food is still prepared, which could be posing a risk to children until colder food is preferred during the warmest months in Beijing City (June, July, and August), but additional studies will have to examine these factors. These observations suggest that programs aiming to educate parents about burn avoidance and treatment may be most useful in spring when children are at the highest risk. As shown above, knowledge of burn prevention was an independent risk factor for burn severity. The majority of pediatric patients lived in cities with an urban to rural residence ratio of 1.68:1; rural patients were overrepresented in this sample according to the 2010 census (urban/rural ratio of 6.14:1) [22], suggesting that children living in rural areas are at higher risk for burns, with an odds ratio of about 3.65 (6.14/1.68). Analysis of housing types also led to a similar conclusion. In rural residences, in particular bungalows (simple and crude house), children may more easily come into contact with fire and hot pots.

In the present study, most burns were mild or moderate, but more pediatric patients with TBSA <15 % were found compared with values previously reported for Sichuan Province [4]. Following burn injury, only 46 % of pediatric patients received appropriate first-aid treatment; the majority received no or inappropriate treatment, resulting in increased burn severity, infection, and poor prognosis. This finding reflects a serious lack of burn treatment knowledge among children’s guardians. In 2005, Cuttle et al. reported that more than 20 min of cold water treatment following burn injury could significantly decrease hospitalization duration; however, only 12.1 % of the pediatric patients assessed here received this treatment [23]. Therefore, improvement of first-aid education for parents/guardians may significantly improve burn prognosis in this population. General hospitals in this region had no department specialized for burns, but admitted the majority of pediatric patients (62 %). A total of 81 % of pediatric patients received no further cold water treatment in such hospitals, which was inappropriate. Therefore, emergency and community physicians also require further education about appropriate burn treatment [23, 24].

In this study, the majority of parents/guardians had no high school degree, and many were farmers or migrant workers. When burns occurred, the majority of children were being supervised by mothers and grandparents. However, most guardians had no or limited knowledge of burn prevention. Therefore, public education for burns prevention should be targeted at populations with low-education levels, and aim to improve the knowledge of common caregivers (mothers and grandparents). For instance, enhanced education should be provided to parents or designated care givers about burn injury, preferentially before child birth. This should encompass the dangers of burns, emergency treatments and first aid protocols, necessity to reach out to a special burn hospital as soon as possible, and keeping children away from heat sources, especially during cooking. Educational level was also shown to be an independent risk factor for burn severity, indicating that public authorities should promote education at large to decrease burn severity.

However, as this study included only 400 patients in one year, whether these findings regarding demographic characteristics of burn patients and their parents/guardians apply to a broader population will require further studies. The impact of targeted education strategies will also require careful monitoring to examine the tangible effects on burn injury in the targeted populations.

The four major Beijing’s hospitals assessed here all have specialized pediatric burn departments. Injured children would be transferred to these centers after primary care by local health centers. This may constitute a selection bias, as children with mild burns might be missed from our analysis. This is a limitation of this study. Another limitation is that a strict follow-up was not implemented, due to the retrospective nature of the study. In addition, only patients alive on discharge were included, probably leading to a selection bias. Nevertheless, we wanted to include patients that were burned by everyday hazards, because patients dying from their burns are usually burned by more severe events such as burning house. Second, patients at risk of dying from their burns are usually treated at the intensive care unit, not at the burn department. Therefore, we could have missed a number of patients. A small number of patients (*n* = 39) were lost to follow-up, which could affect the results of the multivariate analysis of the pigmentation and scar outcomes. Finally, the questionnaire about first aid knowledge was a self-made questionnaire only superficially examining the first aid knowledge of the parents/guardians, and the findings might not be fully comparable with previous reports.

## Conclusions

The current study identified potential risk factors for pediatric burns in Beijing City, including male gender, age under three years, rural residence, and low parental education level. Burns occurred most often during meal preparation times, and scalding was the leading cause. Spring and autumn were the high-risk seasons. The most common anatomical parts involved were head and neck along with low and upper extremities. Burns occurred most frequently in children supervised by mothers and grandparents. Following the burn, 54 % and 81 % of patients did not receive correct emergency and initial treatment, respectively.

Although the establishment of risk factors will require further analysis, our findings suggest that targeting education to parents/guardians and attending clinicians may reduce burn incidence and improve pediatric patient care following burns.

## Additional file

Additional file 1: Raw data of epidemiology in pediatric burned patients of Beijing City. (XLSX 64 kb)

## Abbreviations

TBSA: Total body surface area
